# Massive Retrosternal Thyroid Swelling: A Supraglottic Solution

**DOI:** 10.7759/cureus.107351

**Published:** 2026-04-19

**Authors:** Keerty Sharma, Munira Sidhpurwala, Ranju Singh, Jyoti Kumari, Arshpreet S Grewal

**Affiliations:** 1 Anesthesiology and Critical Care, Lady Hardinge Medical College, New Delhi, IND

**Keywords:** awake fibreoptic intubation (afoi), impossible fona, massive retrosternal thyroid swelling, severe tracheal compression, supraglottic airway device

## Abstract

Massive thyroid swellings with retrosternal extension pose significant airway challenges due to tracheal compression, deviation, and distortion of normal anatomy. Awake fiberoptic bronchoscopy is the gold standard for anticipated difficult airways; however, failure of this technique, particularly when front-of-neck access (FONA) is not feasible, can rapidly lead to a life-threatening airway emergency. We report the management of a 68-year-old American Society of Anesthesiologists (ASA) physical status III female patient with a large retrosternal thyroid swelling causing positional respiratory distress, scheduled for total thyroidectomy. Imaging revealed a sizeable thyroid mass with significant tracheal deviation and compression. Awake fiberoptic nasal intubation under topical anesthesia and conscious sedation was attempted while maintaining spontaneous ventilation but failed due to poor visualization of the glottis. As invasive airway access was technically challenging because of the mass, a supraglottic airway device (i-gel®) was inserted. The device restored effective oxygenation and served as a stable conduit for fiberoptic-guided tracheal intubation, allowing successful endotracheal tube placement beyond the compressed tracheal segment. Surgery proceeded uneventfully. An elective tracheostomy was performed postoperatively in view of suspected tracheomalacia, and the patient made an uncomplicated recovery.

When awake fiberoptic intubation fails and FONA is not feasible, the use of a supraglottic airway device as a conduit for fiberoptic-guided intubation provides a safe and effective alternative. This case highlights the importance of a structured, adaptable airway strategy in patients with massive retrosternal thyroid swellings.

## Introduction

Large neck swellings such as goiters and thyroglossal cysts often pose significant challenges in airway management. As these masses enlarge, they may distort or invade the tracheal lumen, along with progressive compression of adjacent structures, leading to symptoms of airway compromise. This anatomical distortion may lead to difficulty in breathing and also increase the likelihood of difficulty during tracheal intubation [1,2].

Awake fiberoptic bronchoscopy (FOB) is widely regarded as the gold standard for securing the airway in patients with anticipated difficult intubation due to such pathology [3]. However, in certain situations, such as when patient cooperation is limited, airway secretions obscure visualization, or distorted anatomy hinders the passage, awake FOB may fail. Airway management becomes even more complex when front-of-neck access (FONA) is not feasible due to mass location or retrosternal extension, eliminating a key rescue option in the event of airway loss. Even though supraglottic devices (SGDs) play a minimal role in tracheal compression and deviation pathologies, as airway obstruction is beyond the glottic opening, they nonetheless help in scenarios where upper airway obstruction occurs due to sedation in achieving a better laryngeal view [4]. We wish to share a case of thyromegaly with retrosternal invasion, which was managed successfully. An SGD was used as a conduit for fiberscopy, proving to be an effective alternative strategy for ensuring safe airway control in large neck swelling.

## Case presentation

**Figure 1:**
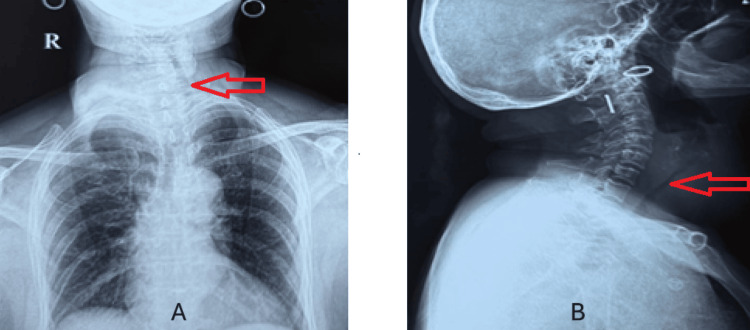
(A) Chest X-ray AP view showing tracheal deviation (red arrow). (B) X-ray neck lateral view showing tracheal compression and enlarged thyroid in the lateral view (red arrow).

**Figure 2:**
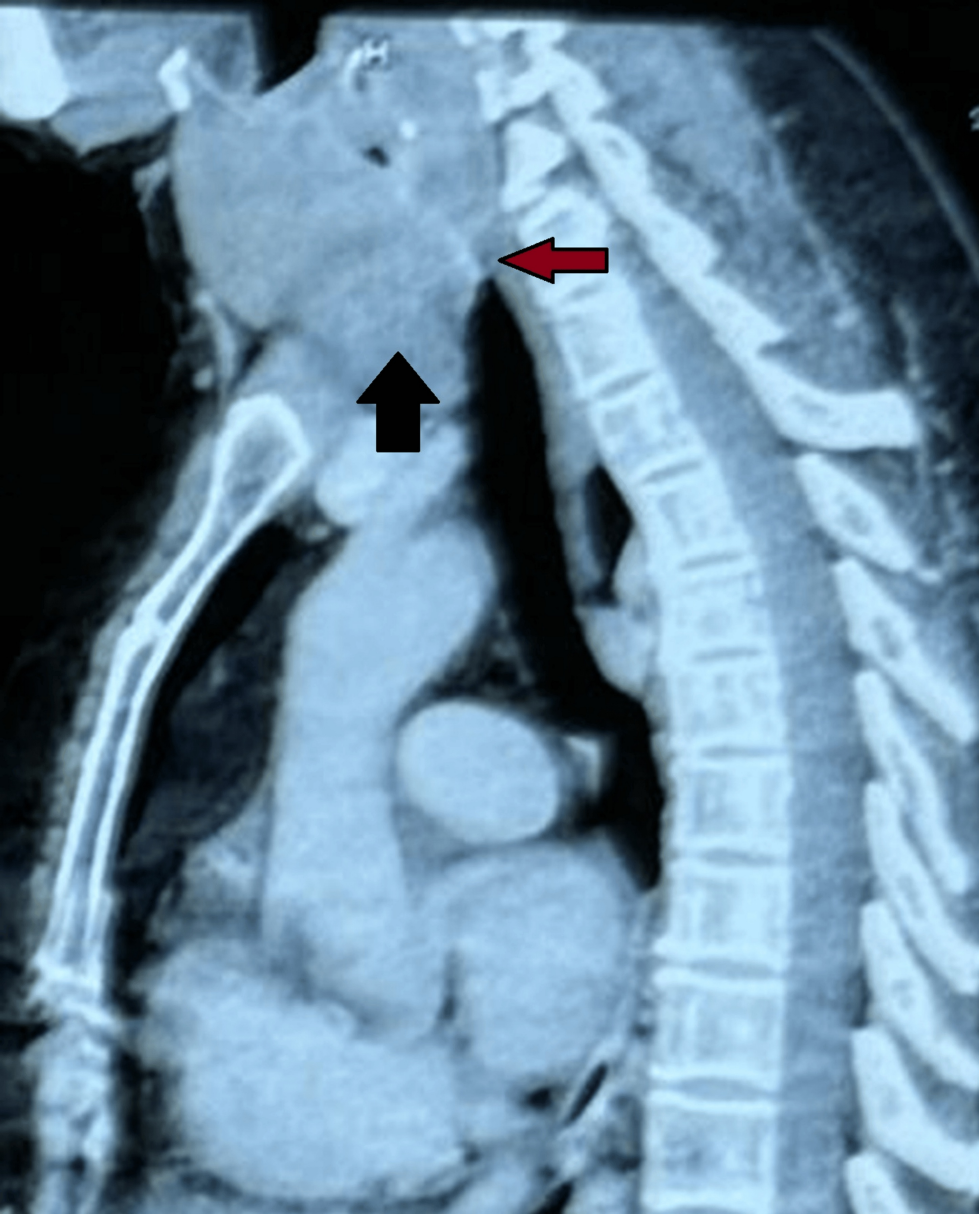
Sagittal reconstructed CT image showing a large retrosternal thyroid swelling extending into the superior mediastinum (black arrow), causing anteroposterior tracheal compression and deviation (red arrow).

Our patient was a 68-year-old American Society of Anesthesiologists Physical Status (ASA PS) - III (uncontrolled hypertensive and controlled hypothyroid) woman with a BMI of 32.5 kg/ m^2^, presenting with gradually increasing swelling in front of the neck for 2.5 months and scheduled for total thyroidectomy. The insidious and gradually progressive swelling was associated with breathlessness in the supine position. There was no history of dysphagia or dyspnea in the sitting and lateral position. On examination, swelling was 10 x 6 cm, firm, non-tender, with retrosternal extension. Airway assessment revealed an edentulous patient with sunken cheeks, no gross facial deformity, B/L patent nares, and modified Mallampati (MMP) class III. X-ray soft tissue head and neck antero-posterior and lateral view suggests tracheal deviation towards the left and compression, respectively (Figure 1). Contrast-enhanced computed tomography neck and chest revealed an ill-defined homogenously enhancing mass of size 6.0 x 6.7 x 9.4 cm in the right lobe of the thyroid gland extending from infrahyoid up to the clavicular region and pushing the trachea to the contralateral side and compressing it, and the anteroposterior diameter at the level of criocoid cartilage is 6.42 mm (Figure 2). Laterally, the lesion abutted the right internal jugular vein with no signs of hemorrhage, necrosis, or calcification within the lesion. Fine needle aspiration cytology revealed a picture of lymphocytic thyroiditis. The patient developed respiratory distress the evening before surgery, and a call was sent to the ICU. She was shifted to the emergency OT for urgent airway management. The patient was tachypneic (26 - 28 breaths/min), with a saturation of 90 - 92% on room air. Our airway plan A was awake flexible videoscope/fiberscopy-guided intubation under topicalization and conscious sedation, plan B was SGD insertion under topicalization and using it as a conduit for fiberscopy and intubation, and plan C was rescue tracheostomy. In the OT, ASA standard monitors (ECG, pulse oximeter, NIBP, and temperature (skin)) were attached, inj. glycopyrrolate 0.2 mg i.v. was administered, and xylometazoline nasal drops (0.1%W/V) were instilled in both nostrils. The patient's airway was topicalized with 4 mL of 4% lignocaine nebulization and lignocaine spray (10%). The patient was pre-oxygenated with 100% oxygen (end tidal oxygen (ETO2) >90%), and para-oxygenation was continued with nasal prongs at 15 L/min. Inj. midazolam 0.5 mg i.v. and inj. fentanyl 20 μg i.v. was given for sedation 10 minutes prior. A flexible videoscope (Karl Storz) of 5.0 mm was loaded with a 6.0 mm ID cuffed endotracheal tube (ETT) and was nasally inserted and advanced with the intermittent SAGO (Spray as You Go) technique. However, vocal cords could not be visualized. Unfortunately, the patient developed a pressure response, and sedation was augmented with aliquots of fentanyl (10 μg), infusion of dexmedetomidine (0.7 μg/kg/hour) & sevoflurane (2-2.5%) in oxygen, ensuring spontaneous breathing at all times. After three unsuccessful attempts with fiberscopy, we switched to plan B; i-gel size # 4 was inserted and confirmed. The fiberscope was advanced, negotiated with some difficulty beyond the level of tracheal compression, and ETT was then railroaded over the FOB. Regrettably, we had to cut the inflation line of ETT while removing the i-gel. Fortunately, there were no leaks and ventilatory insufficiency even with size #6.0 ETT with uniflated cuff due to severe tracheal compression. After securing the airway, inj. propofol (100 mg i.v.), inj. fentanyl 100 μg i.v., and inj. rocuronium (40 mg i.v.) were administered, and total thyroidectomy with cervical extraction of retrosternal extension was performed. The intraoperative course was uneventful. Tracheostomy was done at the end in view of suspected tracheomalacia, and the patient was shifted to the ICU for further management. She was weaned off over a period of a week and decannulated on the eighth post-operative day. Further hospital stay was uneventful, and she was discharged on the 10th postoperative day.

## Discussion

Airway management in patients with massive neck swellings remains one of the most challenging scenarios encountered by anesthesiologists. Large thyroid swellings and midline cystic lesions can cause varying degrees of tracheal deviation, compression, and narrowing, which complicate visualization and instrumentation of the airway [2]. Retrosternal extension further exacerbates these challenges by restricting neck mobility and eliminating the option of FONA, thereby narrowing the margin for error during airway interventions. SGDs have a minimal role in tracheal compression pathologies because compression is past the glottic opening [5]. Awake FOB under topicalization is done routinely, but a rigid bronchoscope and video stylet are suitable alternatives. 

Awake FOB is conventionally the preferred method for securing the airway in anticipated difficult airway situations because it enables spontaneous ventilation and dynamic assessment of airway patency [6]. Distorted airway anatomy, excessive secretions, bleeding, anxiety, or inadequate topical anesthesia, exaggerated pressure response, and desaturation can reduce the likelihood of successful fiberscopy and intubation. When awake, fiberscopy fails, and rescue technique FONA is impossible due to anatomical obstruction by a large mass, the risk of “cannot intubate, cannot oxygenate” (CICO) increases drastically. Such scenarios are worsened by the fact that the cricothyroid membrane in patients with thyromegaly is not easily visualized by use of ultrasound.

In such high-risk settings, a supraglottic airway device may serve as a valuable adjunct. Beyond its role in ventilation, the laryngeal mask airway (LMA) can function as a stable conduit for fiberscopy-guided tracheal intubation. It allows restoration of oxygenation, improves airway alignment, and provides a protected channel for the bronchoscope, thereby increasing the likelihood of successful endotracheal tube placement. Maria et al. concluded that the use of i-gel might result in a better intubation success rate with flexible scope-guided intubation [7]. Michalek et al. in an RCT reported the shortest intubation and insertion time with i-gel with a 100% success rate and better oxygenation [8]. There is limited literature available that suggests SGD-assisted flexible fiberscopy intubation as a lifesaving alternative in scenarios where traditional awake techniques fail, and invasive access is contraindicated or impossible [9,10]. We also suggest intubating SGDs like LMA Blockbuster and AMBU AuraGAIN so that an appropriate size cuffed ETT can be railroaded without damaging the inflation line, as maintaining two airway devices in situ during thyroid surgery/head & neck surgery can be cumbersome. Airway access is often limited because the patient’s head is covered with surgical drapes, and one of the surgical assistants typically stands at the head end of the patient. Our case highlights the unusual but successful use of SGDs in conditions of external tracheal compression. We also emphasize the importance of having a tiered, well-planned airway strategy, tailored to the patient’s anatomical constraints.

## Conclusions

When awake FOB fails, and FONA is not feasible, the use of SGDs as a conduit for fiberscopy-guided intubation offers a practical, safe, and effective solution. This technique not only restores oxygenation but also provides a controlled pathway for advanced airway instrumentation. Our experience underscores the value of supraglottic devices as a critical tool in airway management of large neck swellings with tracheal compression.
